# The preoperative hemoglobin, albumin, lymphocyte, and platelet score as a prognostic Indicator for survival in gastric and colorectal cancer patients

**DOI:** 10.3389/fnut.2026.1892588

**Published:** 2026-08-20

**Authors:** Beijia Zhou, Junxuan Chen, Chen Chen, Qingqing Deng, Yanjun Song, Xiaotian Chen, Tingting Tao

**Affiliations:** 1 Department of Clinical Nutrition, Nanjing Drum Tower Hospital, The Affiliated Hospital of Nanjing University Medical School, Nanjing, Jiangsu, China; 2 The Affiliated Drum Tower Hospital Clinical College of Xuzhou Medical University, Xuzhou, Jiangsu, China; 3 Department of General Surgery, Nanjing Drum Tower Hospital, The Affiliated Hospital of Nanjing University Medical School, Nanjing, Jiangsu, China

**Keywords:** colorectal cancer, gastric cancer, HALP score, nutritional status, prognosis

## Abstract

**Background:**

Gastric and colorectal cancers have high postoperative recurrence despite curative surgery. Current preoperative nutritional assessments often do not fully integrate immune integration and objectivity. The primary objective was to explore preoperative HALP—a composite metric of hemoglobin, albumin, lymphocyte, and platelet values—and postoperative prognosis within the observed follow-up period in gastric and colorectal cancer.

**Methods:**

This prospective cohort study enrolled patients with gastric or colorectal cancer (aged ≥18 years, stage I-III, without neoadjuvant therapy) between May 2021 and June 2022. Preoperative laboratory data within 24 h of admission were collected, and HALP scores were calculated and dichotomized into low and high groups using the established cutoff of 16.81. Patients underwent quarterly postoperative follow-up until May 2025, with overall survival (OS) and progression-free survival (PFS) as endpoints, and statistical analyses included Kaplan–Meier survival curves and Cox regression models.

**Results:**

The study cohort (median age 65 years; 66.8% male) included 75 gastric cancer patients (40.1%) and 112 colorectal cancer patients (59.9%), with 31 patients (16.6%) categorized in the low-HALP group. In both gastric and colorectal cancers, low-HALP patients had significantly inferior OS (log-rank *p* = 0.0056 and 0.0117, respectively), with a non-significant trend toward worse PFS in both subgroups. Multivariable Cox regression confirmed HALP as an independent predictor of OS/PFS (OS HR = 0.138, *p* = 0.000; PFS HR = 0.415, *p* = 0.009) in gastric and colorectal cancers. Critically, after TNM stage adjustment, Low-HALP remained associated with worse survival specifically in Stage III patients (both log-rank *p* < 0.05).

**Conclusion:**

Low preoperative HALP independently predicted poor prognosis in gastric and colorectal cancers during follow-up, particularly in Stage III disease, supporting its use as a complementary marker reflecting the synergistic cascade of malnutrition, inflammation, and immunosuppression linked to tumor aggressiveness.

## Introduction

Gastrointestinal malignancies account for over 25% of global cancer incidence, with their epidemiological trend continuing to rise persistently. According to GLOBOCAN database for 2020 (1), gastric cancer alone caused 1.089 million new cases and 769,000 deaths worldwide, while colorectal cancer—the third most prevalent malignancy globally—ranks as the second leading cause of cancer-related mortality (2, 3). Collectively, these cancers impose a substantial disease burden on global healthcare systems. Radical resection remains the cornerstone of curative treatment for gastric and colorectal cancer. However, despite advances in diagnostic and therapeutic modalities – particularly laparoscopic techniques and early screening programs – postoperative recurrence affects 21.8–50% of gastric cancer patients (4) and approximately 45% of colorectal cancer patients (5), with most recurrences occurring within 2 years postoperatively and often leading to rapid disease progression and lethality. Consequently, evaluating the prognosis subsequent to radical resection for gastric and colorectal carcinoma is critical for directing treatment strategies and follow-up protocols.

Preoperative malnutrition is highly prevalent in gastrointestinal cancer patients, primarily due to tumor-induced reduced food intake and impaired digestive/absorptive function. Moderate-to-severe malnutrition affects approximately 35% of colorectal cancer patients and up to 60% of gastric cancer patients preoperatively (6, 7), and it has been well established as a key determinant of postoperative complications, poor recovery, and reduced overall survival. To address this, validated tools such as the NRS 2002, Prognostic Nutritional Index (PNI), and Patient-Generated Subjective Global Assessment (PG-SGA) are currently used for preoperative nutritional risk screening and malnutrition diagnosis in these patients (8, 9). However, these tools often require subjective assessment (e.g., PG-SGA) or fail to integrate immune status with nutritional markers, limiting their utility in comprehensively evaluating perioperative risk.

In recent years, the HALP score—a novel immunonutritional biomarker combining hemoglobin (H), albumin (A), lymphocyte count (L), and platelet count (P)—has emerged as a promising prognostic indicator in oncology. By integrating hematological (hemoglobin, platelets), nutritional (albumin), and immune (lymphocytes) parameters, HALP offers a holistic reflection of a patient’s systemic status. For instance, Xu et al. (10) demonstrated its prognostic value in pancreatic cancer patients undergoing curative surgery: with a cutoff value of 44.56, patients with low HALP scores had significantly shorter overall survival (OS) and recurrence-free survival (RFS) than those with high scores. Similar prognostic significance has been reported in lung (11), bladder (12), and breast cancer (13). However, few prospective studies have systematically evaluated HALP in both gastric and colorectal cancer within the same cohort, and its differential prognostic impact across tumor types and TNM stages remains unclear.

This prospective cohort study aims to investigate the association between preoperative HALP scores and postoperative prognosis within the observed follow-up period in gastric cancer and colorectal cancer patients undergoing curative surgery, and to assess HALP as a potential objective biomarker to guide prognostic evaluation and nutritional intervention.

## Methods

### Patients

Between May 2021 and June 2022, consecutive patients with primary gastric or colorectal cancer were enrolled from the Department of General Surgery at Nanjing Drum Tower Hospital. Eligible participants were aged over 18 years, had not received preoperative adjuvant radiotherapy or chemotherapy, were admitted for radical surgery, and had a pathological diagnosis confirming stage I-III disease according to the American Joint Committee on Cancer (AJCC) staging system. Exclusion criteria additionally included: (1) severe hepatic or renal insufficiency before surgery; and (2) acute infection (e.g., sepsis, severe pneumonia, acute biliary tract infection) or active autoimmune disease within 2 weeks prior to surgery. Colorectal cancer cases included both colon and rectal cancers. Given that both colon and rectal cancers share similar underlying biological mechanisms and that preliminary analyses showed no significant interaction between HALP and colorectal subsite, we combined them into a single colorectal cancer group. The study received ethical approval from the Clinical Research Ethics Committee (Approval No. 2022–361-01) and adhered to the principles outlined in the Declaration of Helsinki, and informed consent was obtained from all individual participants included in the study.

### Study design

#### Data collection

Patient information was extracted from the electronic medical records database. Collected variables encompassed demographic characteristics (gender, age), comorbidities (history of hypertension, diabetes), anthropometric measures (preoperative weight, body mass index), nutritional status (NRS 2002 score), tumor characteristics (histological type, AJCC stage), surgical details (type of surgery, duration of surgery), and hospitalization outcomes (length of stay, hospital costs, short-term complications).

#### Laboratory parameters

Laboratory indices measured within 24 h of admission included serum albumin (ALB), total protein, hemoglobin, platelet count, C-reactive protein (CRP), lymphocyte count, leukocyte count, neutrophil count, and cholesterol levels. PNI (Prognostic Nutritional Index), PLR (Platelet-to-Lymphocyte Ratio), and NLR (Neutrophil-to-Lymphocyte Ratio) were derived from routine laboratory parameters. Preoperative laboratory parameters were measured within 24 h of admission, prior to any therapeutic intervention. None of the patients received preoperative blood transfusion, intravenous iron, erythropoietin, or platelet transfusion, ensuring that the HALP score reflected the genuine baseline status without iatrogenic modification.

#### Follow-up protocol

All patients underwent follow-up assessments every 3 months postoperatively until death or the end of the study period (May 31, 2025). Clinically relevant information was collected at each visit according to standard follow-up protocols. The primary endpoints were OS and PFS. Median follow-up time was estimated using the reverse Kaplan–Meier method, with censored observations inverted as events.

#### HALP score calculation

The HALP score was computed according to the formula: HALP = [Hemoglobin (g/L) × Albumin (g/L) × Lymphocyte count (×10^9^/L)]/[Platelet count (×10^9^/L)].

#### Determination of HALP score cutoff

Using X-tile software (version 3.6.1, Yale University), the optimal cutoff value for the HALP score was identified as 16.81 based on overall survival (OS) outcomes. X-tile software automatically performs permutation-based multiple testing correction and provides cross-validation to minimize overfitting. According to this threshold, patients were stratified into low-HALP (≤16.81) and high-HALP (>16.81) groups.

### Statistical analysis

Given the exploratory nature of this study, no formal sample size calculation was performed. We enrolled consecutive eligible patients within a predefined window (May 2021–June 2022). A post-hoc power analysis based on the observed events (25 deaths, 48 progressions) indicated that the final cohort (*N* = 187) had 80% power to detect a HR of approximately 3.0 for OS and 2.3 for PFS (*α* = 0.05, *β* = 0.20), assuming a two-group comparison with the observed group proportions. We acknowledge that subgroup analyses were underpowered, as noted in the Discussion. Continuous variables with normal distribution were compared using unpaired Student’s t-tests, with data presented as mean ± standard deviation (SD). Non-normally distributed continuous variables were summarized as median (interquartile range, IQR) and analyzed via Mann–Whitney U tests. Categorical variables underwent chi-square or Fisher’s exact tests, reported as frequencies (percentages). Survival outcomes were evaluated through Kaplan–Meier curves for OS and PFS, with between-group differences assessed by log-rank tests. Univariate and multivariate Cox proportional hazards regression models identified independent prognostic factors. All analyses utilized IBM SPSS Statistics (v21.0), while figures were constructed with the ‘Forest Map Creation’ module on the Mengte Cloud analytics platform.^1^ Two-sided *p*-values <0.05 defined statistical significance.

## Results

### Baseline characteristics and surgery-related clinical features

Among the 187 patients, 75 had gastric cancer and 112 had colorectal cancer (73 colon, 39 rectal); the latter two were merged for analysis. Baseline characteristics stratified by cancer type are shown in Table 1. Gastric and colorectal cancer patients were largely comparable in demographics, comorbidities, and most laboratory parameters, except that gastric cancer patients had higher NRS 2002 scores and a greater proportion with NRS 2002 ≥ 3 (76.0% vs. 55.4%, *p* = 0.004). Laparotomy was more frequent in gastric cancer (74.7% vs. 12.5%, *p* < 0.001), with earlier stage distribution but longer hospital stays and higher costs (all *p* < 0.01). Mortality at last follow-up was higher in gastric cancer (20.0% vs. 8.9%, *p* = 0.029), whereas complication, recurrence, and adjuvant therapy rates did not differ.

**Table 1:** Baseline characteristics and surgery-related clinical features.

Characteristics	Overall *N* = 187	Gastric cancer *N* = 75	Colorectal cancer *N* = 112	*p*
Age (year)	65.00 (57.00, 73.00)	65.00 (58.00, 71.50)	65.00 (57.00, 74.00)	0.922
Male (*n*, %)	125 (66.84)	56 (74.67)	69 (61.61)	0.063
Height (m)	1.66 (1.60, 1.70)	1.65 (1.60, 1.70)	1.67 (1.60, 1.70)	0.616
Weight (kg)	64.00 (58.00, 70.75)	64.00 (58.00, 70.00)	64.75 (57.00, 72.62)	0.497
BMI (kg/m^2^)	23.60 ± 3.02	23.49 ± 3.00	23.66 ± 3.04	0.706
Diabetes (*n*, %)	23 (12.30)	10 (13.33)	13 (11.61)	0.725
Hypertension (*n*, %)	64 (34.22)	26 (34.67)	38 (33.93)	0.917
NRS 2002 score	3.00 (2.00, 3.00)	3.00 (3.00, 4.00)	3.00 (2.00, 3.00)	0.006*
NRS 2002 ≥ 3 (*n*, %)	119 (63.64)	57 (76.00)	62 (55.36)	0.004*
PNI	47.20 (44.15, 50.45)	47.10 (44.10, 49.35)	47.55 (44.35, 50.92)	0.359
PLR	138.57 (100.21, 200.00)	135.56 (100.00, 201.30)	139.44 (101.98, 200.00)	0.756
NLR	2.11 (1.59, 2.89)	2.08 (1.62, 2.60)	2.20 (1.53, 2.93)	0.416
HALP score	37.88 (22.45, 53.01)	39.47 (25.05, 56.30)	36.72 (22.08, 51.21)	0.592
HALP group				0.820
≤16.81	31 (16.58)	13 (17.33)	18 (16.07)	
>16.81	156 (83.42)	62 (82.67)	94 (83.93)	
Total protein (g/L)	62.62 ± 5.85	61.81 ± 5.94	63.17 ± 5.76	0.121
ALB (g/L)	39.70 (37.55, 41.40)	39.30 (37.40, 41.05)	39.70 (37.68, 41.40)	0.334
CRP (mg/L)	2.50 (2.05, 4.00)	2.30 (2.00, 3.55)	2.70 (2.10, 5.12)	0.082
Hemoglobin (g/L)	128.00 (111.00, 141.00)	130.00 (112.50, 144.50)	127.00 (110.75, 138.00)	0.329
Platelet (×10^9^/L)	218.00 (174.50, 273.50)	218.00 (168.00, 270.00)	218.50 (178.50, 275.75)	0.475
Neutrophils (×10^9^/L)	3.30 (2.60, 4.05)	3.10 (2.60, 3.60)	3.40 (2.77, 4.20)	0.036*
Leucocyte (×10^9^/L)	5.40 (4.70, 6.50)	5.10 (4.40, 6.35)	5.70 (4.80, 6.62)	0.032*
Lymphocyte (×10^9^/L)	1.50 (1.20, 1.85)	1.50 (1.20, 1.85)	1.50 (1.20, 1.83)	0.422
Cholesterol (mmol/L)	4.17 ± 0.86	4.13 ± 0.84	4.20 ± 0.88	0.561
Operation type (*n*, %)				<0.001**
Laparoscope	117 (62.57)	19 (25.33)	98 (87.50)	
Laparotomy	70 (37.43)	56 (74.67)	14 (12.50)	
Tumor stage (AJCC)				<0.001**
I	44 (23.53)	30 (40.00)	14 (12.50)	
II	70 (37.43)	23 (30.67)	47 (41.96)	
III	73 (39.04)	22 (29.33)	51 (45.54)	
Duration of surgery (min)	205.00 (172.50, 240.00)	200.00 (180.00, 240.00)	205.00 (170.00, 235.00)	0.559
Hospitalization days (d)	16.00 (14.00, 19.00)	17.00 (15.00, 19.00)	15.00 (13.00, 18.00)	0.006*
Post-operative length of stay (d)	10.00 (9.00, 12.00)	11.00 (10.00, 13.00)	9.00 (8.00, 11.00)	<0.001**
Hospital costs (RMB)	67332.54 (61828.95, 76216.55)	74158.90 (67349.95, 88072.41)	64199.68 (60280.15, 68823.99)	<0.001**
Short-term complications (*n*, %)				0.243
Intestinal obstruction	3 (5.77)	1 (4.35)	2 (6.90)	
Cutaneous infection	14 (26.92)	3 (13.04)	11 (37.93)	
Abdominal bloating	8 (15.38)	6 (26.09)	2 (6.90)	
Abdominal infection	8 (15.38)	4 (17.39)	4 (13.79)	
Pulmonary infection	7 (13.46)	4 (17.39)	3 (10.34)	
Fungal infection	2 (3.85)	1 (4.35)	1 (3.45)	
Urinary tract infection	2 (3.85)	1 (4.35)	1 (3.45)	
Fat liquefaction	2 (3.85)	0 (0.00)	2 (6.90)	
Abdominal haemorrhage	1 (1.92)	1 (4.35)	0 (0.00)	
Upper respiratory infection	1 (1.92)	0 (0.00)	1 (3.45)	
Biliary fistula	1 (1.92)	0 (0.00)	1 (3.45)	
Gastroparesis	1 (1.92)	1 (4.35)	0 (0.00)	
Leakage of milk curd	1 (1.92)	1 (4.35)	0 (0.00)	
Drainage tube port infection	1 (1.92)	0 (0.00)	1 (3.45)	
Postoperative recurrence (*n*, %)				0.932
Yes	48 (25.67)	19 (25.33)	29 (25.89)	
No	139 (74.33)	56 (74.67)	83 (74.11)	
Postoperative adjuvant therapy (*n*, %)				0.531
Yes	95 (50.80)	36 (48.00)	59 (52.68)	
No	92 (49.20)	39 (52.00)	53 (47.32)	
Death (*n*, %)				0.029*
Yes	25 (13.37)	15 (20.00)	10 (8.93)	
No	162 (86.63)	60 (80.00)	102 (91.07)	

*indicated for *p* < 0.05; NRS 2002: nutritional risk screening 2002; BMI: body mass index; ALB: albumin; CRP: C-reactive protein; PNI: prognostic nutritional index; PLR: platelet-to-lymphocyte ratio; NLR: neutrophil-to-lymphocyte ratio; AJCC: American Joint Committee on Cancer; RMB: Renminbi (Chinese yuan).

Using the HALP cutoff of 16.81, 31 patients (16.6%) were classified as low-HALP and 156 (83.4%) as high-HALP. Baseline characteristics by HALP group are provided in Supplementary Table 1. Low-HALP patients were significantly older, had lower weight and BMI, worse hematological parameters (lower hemoglobin, albumin, and lymphocytes; higher platelets), and a higher prevalence of hypertension (51.6% vs. 30.8%, *p* = 0.025) compared to the high-HALP group (Supplementary Table 1).

### Follow-up duration

As of the data cutoff date (May 31, 2025), the median follow-up duration for the entire cohort was 37.0 months (interquartile range: 34.0–42.0 months), calculated using the reverse Kaplan–Meier method. During this period, the median overall survival and median progression-free survival were not reached due to the low event rate, indicating favorable interim outcomes in a substantial proportion of patients. The 1-year and 3-year OS rates were higher in high-HALP versus low-HALP patients (98.7% vs. 86.1 and 95.5% vs. 67.0%, respectively). Similarly, PFS rates at 1/3 years favored the high-HALP group (92.9% vs. 82.8 and 76.1% vs. 58.0%).

### Survival curves of OS and PFS stratified by HALP score within each cancer type

**Figure 1:**
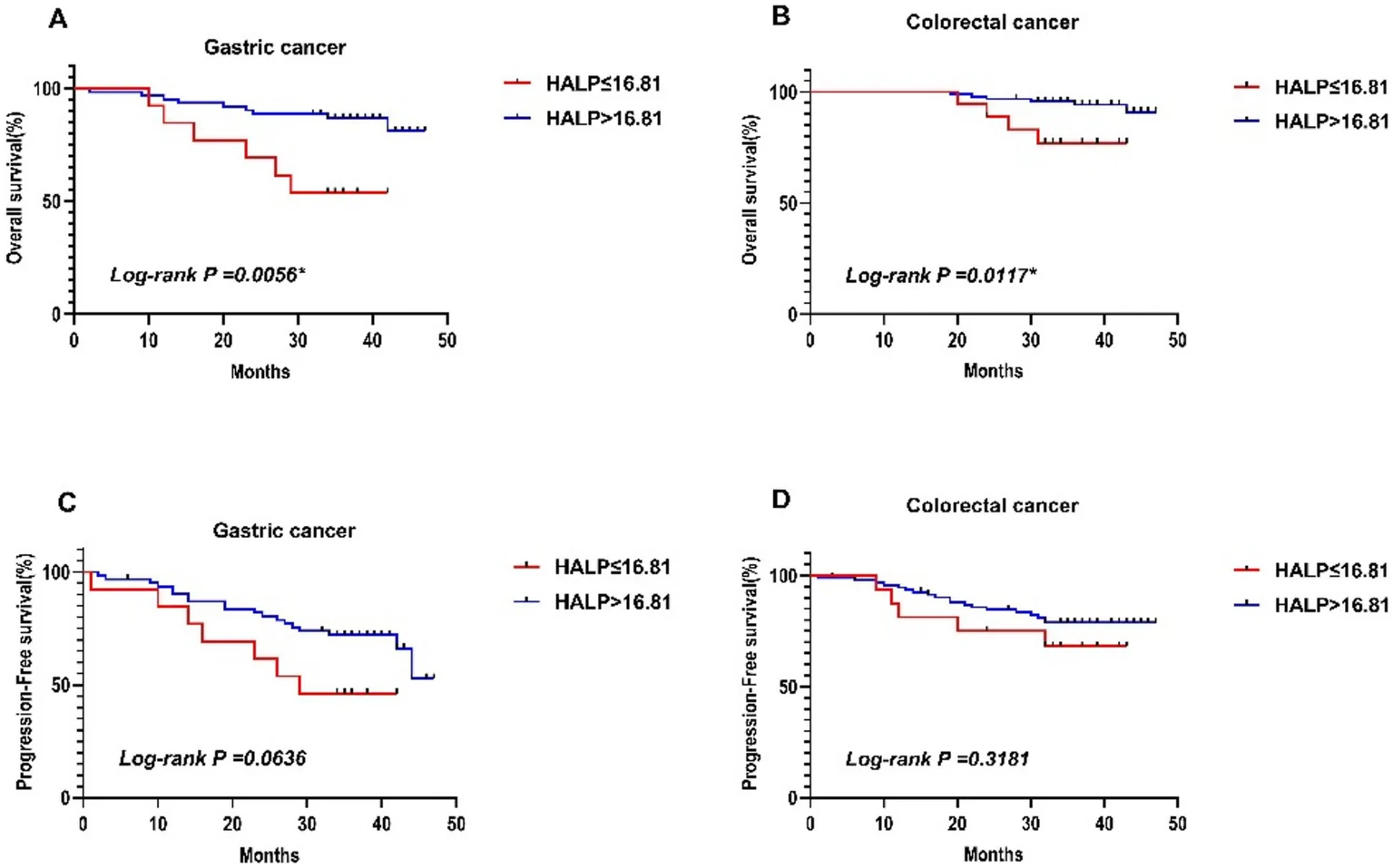
Kaplan–Meier estimates of OS and PFS stratified by HALP score within each cancer type. **(A)** OS for gastric cancer; **(B)** OS for colorectal cancer; **(C)** PFS for gastric cancer; **(D)** PFS for colorectal cancer.

Kaplan–Meier analyses stratified by cancer type revealed distinct survival patterns between HALP groups (Figure 1). For overall survival, patients with a high HALP score (>16.81) exhibited significantly better OS than those with a low HALP score in both gastric cancer (log-rank *p* = 0.0056) and colorectal cancer (log-rank *p* = 0.0117). In contrast, for progression-free survival, the differences between high- and low-HALP groups did not reach statistical significance in either gastric cancer (log-rank *p* = 0.0636) or colorectal cancer (log-rank *p* = 0.3181), although a trend toward better PFS in the high-HALP group was observed in gastric cancer patients.

### Univariate and multivariate cox regression analysis of OS predictors in gastric and colorectal cancers

Univariate Cox regression identified BMI, PNI, PLR, HALP group, cancer type, operation type, surgical duration, postoperative length of stay, and recurrence as significant predictors of overall survival in gastric and colorectal cancer patients (all *p* < 0.05). After adjusting for age, gender, BMI, TNM stage, operation type, PNI, PLR, and adjuvant therapy, multivariate Cox analysis established high HALP as an independent protective factor for overall survival (HR = 0.138, 95% CI: 0.054–0.349; *p* < 0.001). Conversely, prolonged postoperative hospitalization (HR = 1.125, 95% CI: 1.054–1.202; *p* = 0.000), duration of surgery (HR = 1.012, 95% CI: 1.005–1.018; *p* < 0.001) and recurrent metastases (HR = 5.734, 95% CI: 2.428–13.538; *p* < 0.001) were identified as significant risk factors for reduced survival (Table 2).

**Table 2:** Univariate and multivariate analysis of factors associated with OS.

	Univariate analysis		Multivariate analysis	
Factors	HR (95% CI)	*p*	HR (95% CI)	*p*
Age	1.022 (0.986, 1.060)	0.234		
Sex (female vs. male)	1.403 (0.630, 3.125)	0.407		
BMI	0.847 (0.732, 0.980)	0.025*		
NRS 2002 ≥ 3	2.560 (0.959, 6.832)	0.061		
Diabetes (yes vs. no)	1.341 (0.460, 3.907)	0.591		
Hypertension (yes vs. no)	1.091 (0.482, 2.470)	0.834		
PNI	0.864 (0.791, 0.943)	0.001*		
PLR	1.003 (1.001, 1.005)	0.018*		
NLR	1.108 (0.927, 1.324)	0.261		
HALP group (high vs. low)	0.238 (0.106, 0.535)	0.001*	0.138 (0.054, 0.349)	0.000**
Cancer type (colorectal cancer vs. gastric cancer)	0.399 (0.179, 0.889)	0.025*	0.327 (0.140, 0.763)	0.010*
Operation type (laparotomy vs. laparoscope)	2.670 (1.198, 5.949)	0.016*		
Tumor stage (AJCC)		0.303		
II vs. I	1.856 (0.502, 6.858)	0.354		
III vs. I	2.609 (0.743, 9.158)	0.135		
Duration of surgery	1.010 (1.003, 1.016)	0.004*	1.012 (1.005, 1.018)	0.001*
Post-operative length of stay	1.127 (1.063, 1.195)	0.000**	1.125 (1.054, 1.202)	0.000**
Short-term complications (yes vs. no)	1.174 (0.782, 3.876)	0.175		
Postoperative Recurrence (yes vs. no)	4.865 (2.182, 10.849)	0.000**	5.734 (2.428, 13.538)	0.000**
Postoperative adjuvant therapy (yes vs. no)	1.724 (0.762, 3.903)	0.191		
Total protein	0.933 (0.870, 1.002)	0.056		
Cholesterol	0.668 (0.418, 1.068)	0.092		
CRP	1.006 (0.991, 1.021)	0.433		
Leucocyte	0.718 (0.330, 1.565)	0.405		
Neutrophils	1.145 (0.940, 1.394)	0.180		

*indicated for *p* < 0.05; **indicated for *p* < 0.001; HR: hazard ratios; CI: confidence interval; BMI: body mass index; ALB: albumin; CRP: C-reactive protein; PNI: prognostic nutritional index; PLR: platelet-to-lymphocyte ratio; NLR: neutrophil-to-lymphocyte ratio; AJCC: American joint committee on cancer.

### Subgroup analyses according to cancer type of OS

**Figure 2:**
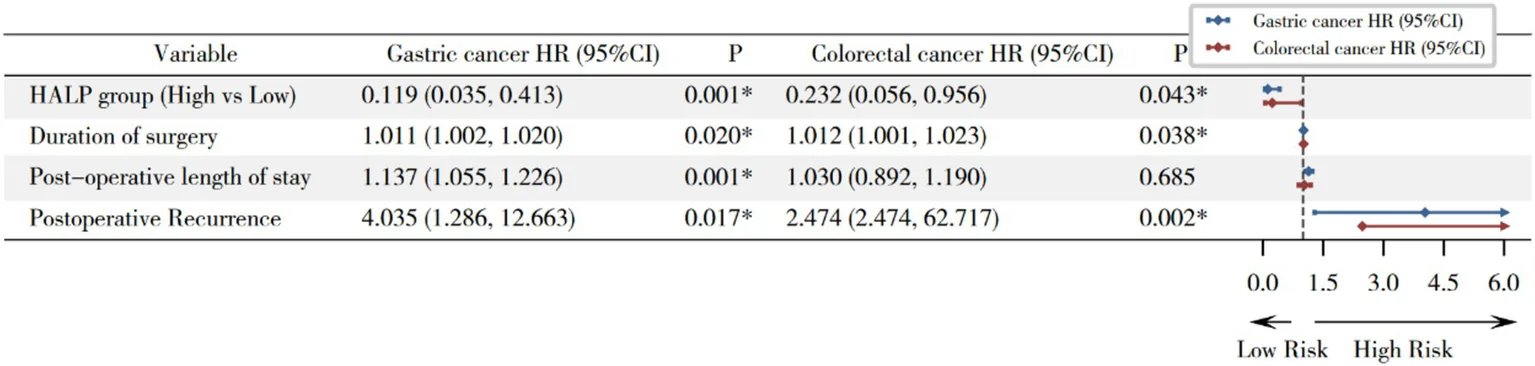
Subgroup analyses and forest plot of gastric cancer and colorectal cancer. *indicated for *p* < 0.05; HR: azard ratios; CI: confidence interval.

Subgroup analyses demonstrated that elevated preoperative HALP was a protective factor for OS in both gastric cancer and colorectal cancer patients (*p* < 0.05) in Figure 2. Conversely, prolonged operative duration and recurrent metastases independently predicted reduced OS. Postoperative hospitalization exerted differential effects by cancer type, significantly increasing mortality risk only in gastric cancer patients (*p* < 0.05).

### Univariate and multivariate cox regression analysis of PFS predictors in gastric and colorectal cancers

Univariate analysis identified elevated HALP, PNI, and total protein as protective factors for PFS, whereas laparotomy and prolonged hospitalization after surgery predicted reduced PFS (*p* < 0.05). However, postoperative adjuvant therapy unexpectedly appeared as a risk factor for reduced PFS (HR = 2.439, 95% CI: 1.349–4.410, *p* = 0.003). This counterintuitive finding is attributable to confounding by indication, as treatment allocation was strongly associated with advanced TNM stage. After multivariable adjustment including TNM stage, the effect of adjuvant therapy was neutralized. Multivariate analysis incorporating treatment-by-stage interaction terms, adjusted for age, gender, and TNM stage, confirmed postoperative therapy as a protective factor across all stages (*p* < 0.05). Additionally, the effects of HALP score (HR = 0.415, 95% CI: 0.213–0.805; *p* = 0.009) and postoperative length of stay (HR = 1.089, 95% CI: 1.033–1.147; *p* = 0.001) remained independently associated with PFS in the multivariate model (Table 3).

**Table 3:** Univariate and multivariate analysis of factors associated with PFS.

	Univariate analysis		Multivariate analysis	
Factors	HR (95% CI)	*p*	HR (95% CI)	*p*
Age	1.005 (0.981, 1.030)	0.671		
Sex (female vs. male)	0.927 (0.513, 1.674)	0.801		
BMI	0.914 (0.833, 1.004)	0.060		
NRS 2002 ≥ 3	1.706 (0.922, 3.158)	0.061		
Diabetes (yes vs. no)	0.748 (0.297, 1.884)	0.538		
Hypertension (yes vs. no)	0.699 (0.378, 1.294)	0.255		
PNI	0.922 (0.868, 0.980)	0.009*		
PLR	1.001 (0.999, 1.004)	0.192		
NLR	1.027 (0.885, 1.191)	0.729		
HALP group (high vs. low)	0.470 (0.250, 0.884)	0.019*	0.415 (0.213, 0.805)	0.009*
Cancer type (colorectal cancer vs. gastric cancer)	0.620 (0.358, 1.075)	0.089		
Operation type (laparotomy vs. laparoscope)	2.010 (1.159, 3.486)	0.013*		
Tumor stage (AJCC)		0.219		
II vs. I	0.799 (0.367, 1.741)	0.573		
III vs. I	1.395 (0.686, 2.838)	0.358		
Post-operative length of stay	1.080 (1.029, 1.133)	0.002*	1.089 (1.033, 1.147)	0.001*
Short-term complications (yes vs. no)	1.137 (0.629, 2.055)	0.670		
Postoperative adjuvant therapy (yes vs. no)	2.439 (1.349, 4.410)	0.003*	7.854 (2.569, 24.007)	0.000**
Total protein	0.950 (0.903, 0.999)	0.044*		
Cholesterol	0.722 (0.521, 1.000)	0.050		
CRP	1.000 (0.985, 1.015)	0.972		
Leucocyte	0.718 (0.422, 1.222)	0.222		
Neutrophils	1.014 (0.832, 1.234)	0.893		
TNM* therapy				0.090
TNM (2) * therapy			0.226 (0.069, 0.734)	0.013*
TNM (3) * therapy			0.291 (0.096, 0.876)	0.028*

* indicated for *p* < 0.05; **indicated for *p* < 0.001; HR: hazard ratios; CI: confidence interval; BMI: body mass index; ALB: albumin; CRP: C-reactive protein; PNI: prognostic nutritional index; PLR: platelet-to-lymphocyte ratio; NLR: neutrophil-to-lymphocyte ratio; AJCC: American Joint Committee on Cancer.

### Subgroup multivariate cox regression for PFS stratified by cancer type

In the stratified subgroup analysis (Supplementary Table 2), the HALP score was not significantly associated with PFS in either gastric cancer (HR = 0.549, 95% CI: 0.211–1.430, *p* = 0.220) or colorectal cancer (HR = 0.417, 95% CI: 0.162–1.071, *p* = 0.069), although a protective trend favoring high HALP was observed. Adjuvant therapy was significantly associated with improved PFS only in colorectal cancer (HR = 7.115, 95% CI: 2.097–24.145, *p* = 0.002).

### Kaplan–Meier curves for OS and PFS stratified by HALP score across TNM stages

**Figure 3:**
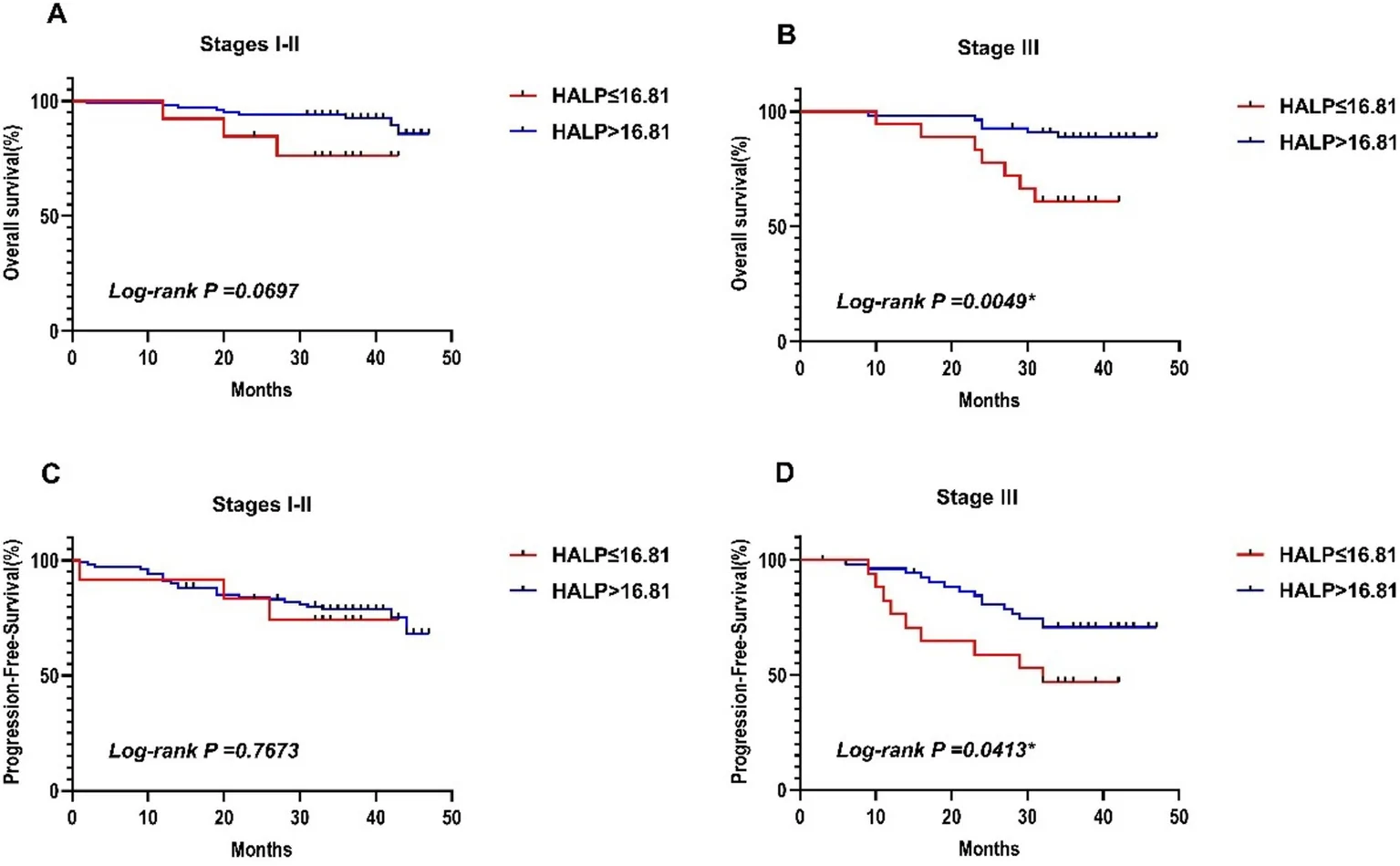
Kaplan–Meier estimates of OS and PFS stratified by HALP score among early-stage (I-II) and advanced-stage (III) patients. **(A)** OS of TNM stage I-II; **(B)** OS of TNM stage III; **(C)** PFS of TNM stage I-II; **(D)** PFS of TNM stage III.

This study examined the prognostic value of HALP levels across TNM stages, categorizing early-stage (I-II) and advanced-stage (III) cancers. As demonstrated in Figure 3, survival analysis revealed significantly reduced OS (log-rank *p* = 0.0049) and PFS (log-rank *p* = 0.0413) in the low-HALP group compared to the high-HALP group among advanced-stage patients. No such survival disadvantage was observed in early-stage cohorts.

## Discussion

In this cohort of 187 gastric and colorectal cancer patients, using an X-tile-derived HALP cutoff of 16.81, Kaplan–Meier analyses demonstrated significantly better OS in the high-HALP group across both cancer types, with a consistent but non-significant trend for PFS; multivariate Cox regression confirmed HALP as an independent predictor of both OS and PFS. Notably, the prognostic impact of HALP was most pronounced in stage III disease, where low HALP remained associated with worse survival even after TNM adjustment, underscoring its value as a complementary risk stratifier in the highest-risk subgroup. The non-significant PFS results in the stratified Kaplan–Meier analyses may be attributable to the limited number of progression events and the relatively short follow-up, which reduced statistical power. Nevertheless, the consistent direction of the hazard ratios across subgroups and the significant results in the overall multivariate Cox model support a potential protective effect of HALP on PFS that merits further investigation in larger cohorts.

The HALP cutoff in this study was derived from the same cohort using X-tile software rather than adopting previously published thresholds. This decision was based on several considerations. First, substantial inter-study variability in HALP cutoff values has been documented across different populations and cancer types, ranging from 2.22 to 62.14, which limits the applicability of a universal threshold (14). Second, we initially attempted to apply previously reported cutoff values from other cohorts to our population, but these thresholds failed to achieve optimal risk stratification, likely due to differences in surgical approaches, baseline nutritional status, and tumor characteristics. To mitigate the risk of overfitting, we employed X-tile software, which incorporates permutation-based multiple testing correction and internal cross-validation to ensure stability of the identified threshold (15).

An incidental observation in univariate PFS analysis identified adjuvant therapy as a risk factor, a paradox explained by non-randomized treatment allocation: adjuvant therapy was given to 75.3% of stage III patients versus 15.9% of stage I and 47.1% of stage II patients. This effect diminished after multivariate adjustment for TNM stage, highlighting the necessity of multivariable correction in observational data. While treatment efficacy is not a primary objective of this study, the observation supports the robustness of the HALP-related conclusions.

Prior investigations have established the prognostic relevance of the HALP score across multiple solid tumor types. A recent systematic review and meta-analysis comprising 30 articles, 34 studies, and 9,389 patients demonstrated that high HALP scores were significantly associated with improved survival outcomes across digestive system cancers, with pooled HRs of 1.762 for OS (95% CI: 1.570–1.977) and 1.444 for PFS (95% CI: 1.068–1.954). In gastric cancer, Aoyama et al. (16) demonstrated that with a cutoff value of 40, HALP served as an independent prognostic indicator for OS in patients undergoing radical resection (HR = 2.679, 95% CI: 1.455–4.934, *p* = 0.002). Their analysis further revealed that the low HALP cohort exhibited higher rates of postoperative complications and required adjuvant chemotherapy more frequently than the high HALP group. Complementing this, another study with 349 patients validated HALP as an independent risk factor for lymph node metastasis in gastric cancer (17).

In colorectal cancer research, accumulating evidence from large-scale retrospective studies has consistently supported the prognostic value of HALP. A well-powered analysis of 1,441 patients demonstrated that low HALP was independently associated with worse PFS and OS, with HALP-based nomograms demonstrating robust predictive performance (18). These findings were further corroborated by independent cohorts: Çağlıyan et al. (19) reported that a HALP score below 23 predicted poor OS in colorectal cancer patients, while Yalav et al. (20), in a cohort of 279 patients undergoing curative resection, identified a cutoff of 15.73 and confirmed HALP as an independent prognostic factor for survival (HR = 0.855, *p* = 0.007). Collectively, these data reinforce the clinical utility of HALP as a reproducible and generalizable prognostic marker in colorectal cancer, complementing the results of the present study. Dagmura et al. (21) further identified HALP and age as promising survival biomarkers in elderly patients following radical resection. Notably, however, a separate investigation found no utility for HALP in the early diagnosis of colorectal cancer metastasis or staging, highlighting its limited value in detecting early-stage disease progression (22). Collectively, these findings align with the present study’s core conclusion that HALP significantly influences postoperative prognosis within the observed follow-up period, particularly OS. This study systematically investigates both gastric and colorectal cancer, reports the role of PFS, and provides a basis for exploring HALP as a prognostic marker in gastric and colorectal cancers. Importantly, it is the first to report a significant association between HALP and prognosis in advanced patients even after adjusting for TNM staging.

The HALP score integrates four readily available laboratory parameters—hemoglobin, albumin, lymphocyte, and platelet counts—each reflecting distinct yet interconnected aspects of host physiology that collectively shape cancer prognosis. Hemoglobin captures tumor-related anemia and subsequent hypoxia, which promotes aggressive tumor behavior and treatment resistance (23, 24); albumin reflects nutritional reserve and systemic inflammatory burden (25); lymphocyte count serves as a proxy for host immunocompetence and antitumor surveillance (26, 27); and platelet count mirrors thrombocytosis driven by tumor-induced inflammatory cytokines, which facilitates angiogenesis and metastatic spread (28). A low HALP score thus signifies the convergence of malnutrition, systemic inflammation, and immune exhaustion into a permissive tumor microenvironment. This integrated signature is consistent with our observation that the prognostic impact of HALP was most pronounced in stage III disease, where the metabolic and immunological demands of advanced tumors are greatest and may overwhelm the host’s compensatory capacity. The progressive decline of HALP scores with advancing disease likely reflects the cumulative burden of these synergistic pathophysiological changes.

While the established TNM staging system, assessing local tumor invasion and distant metastasis, remains foundational for prognostic stratification, it has inherent limitations. TNM demonstrates suboptimal precision in outcome prediction (29) due to its inability to capture individual tumor biology, molecular drivers of progression, or post-therapeutic dynamics (29, 30). In contrast, the HALP composite score—integrating hemoglobin, albumin, lymphocytes, and platelets—may more sensitively capture systemic host determinants that fuel tumor evolution and modulate therapeutic vulnerability. These systemic factors critically determine prognosis in advanced disease, precisely where anatomical TNM staging becomes inadequate. Furthermore, among patients requiring postoperative adjuvant therapy (e.g., chemoradiation, targeted agents), compromised host status reflected by low HALP scores directly impairs treatment feasibility and clinical response. The pronounced prognostic impact of HALP in stage III patients may reflect the greater metabolic and immunological demands of advanced tumors, making patients more vulnerable to malnutrition-inflammation-immunosuppression cascades. In early-stage disease, host reserves may still be sufficient to compensate, thus attenuating the prognostic effect of HALP.

This study adds to the evidence supporting the independent prognostic utility of the HALP score in gastric and colorectal cancer patients undergoing surgery. Calculated from routine, cost-effective blood parameters, HALP provides a clinically accessible biomarker. Preoperative assessment effectively supplements the TNM staging system, particularly by stratifying high-risk advanced-stage patients with inferior prognoses. Future investigations should prioritize evaluating intensified multimodal adjuvant strategies and establishing vigilant surveillance protocols for advanced-stage patients identified as high-risk by low HALP scores.

This investigation has several limitations. The single-center design and relatively limited sample size may restrict generalizability and precluded separate analyses for colon and rectal cancer subtypes. In addition, histological subtypes (e.g., signet-ring cell carcinoma in gastric cancer or mucinous adenocarcinoma in colorectal cancer) were not specifically examined; given their distinct prognostic implications, their absence may have introduced unmeasured confounding, although the limited sample size and low subtype prevalence precluded stratified analyses. Furthermore, the median follow-up of 37.0 months (IQR: 34.0–42.0 months) does not permit a robust assessment of 5-year survival, and the limited number of progression events may have reduced the statistical power of stratified PFS analyses, warranting cautious interpretation of the observed trends. Longer follow-up and multi-center external validation are needed to confirm the optimal HALP threshold and its sustained prognostic impact, and to determine whether its predictive value varies across histological subtypes.

## Conclusion

Within a median follow-up of 37 months, preoperative low HALP was associated with worse overall survival in both gastric and colorectal cancer patients, particularly in stage III disease, and showed a consistent protective trend for progression-free survival that reached significance in the overall multivariate analysis. The HALP score may serve as a complementary biomarker to TNM staging for risk stratification. Larger multi-center studies with longer follow-up are needed to confirm its independent prognostic value.

## Data Availability

The original contributions presented in the study are included in the article/Supplementary material, further inquiries can be directed to the corresponding authors.

## Footnotes

1
https://mengte.pro/forest_plot
