# Supplementary material for: The preoperative hemoglobin, albumin, lymphocyte, and platelet score as a prognostic Indicator for survival in gastric and colorectal cancer patients
Source: Front Nutr. 2026 Aug 20;13:1892588. doi: 10.3389/fnut.2026.1892588 (PMC13538017; doi:10.3389/fnut.2026.1892588)
Supplement: Supplementary file 1 [file Table_1.docx]

**Supplement.1 Baseline Characteristics and Surgery-Related Clinical Features Stratified by HALP Score (≤16.81 vs. >16.81)**

| **Characteristics** | | **Overall**  **N=187** | **Low-HALP group**  **N=31** | **High-HALP group**  **N=156** | ***P*** |
| --- | --- | --- | --- | --- | --- |
| Age (year) | 65.00 (57.00, 73.00) | | 73.00 (67.00, 76.00) | 63.00 (56.00, 71.25) | <0.001 |
| Male (n, %) | 125 (66.84) | | 21 (67.74) | 104 (66.67) | 0.908 |
| Height (m) | 1.65 ± 0.08 | | 1.64 ± 0.08 | 1.66 ± 0.08 | 0.192 |
| Weight (kg) | 64.00 (58.00, 70.75) | | 60.00 (52.75, 66.50) | 65.00 (58.50, 72.00) | 0.006* |
| BMI (kg/m^2^) | 23.60 ± 3.02 | | 22.06 ± 2.47 | 23.90 ± 3.03 | 0.002* |
| Diabetes (n, %) | 23 (12.30) | | 5 (16.13) | 18 (11.54) | 0.681 |
| ALB(g/L) | 39.32 ± 2.96 | | 35.85 ± 3.31 | 40.01 ± 2.34 | <0.001 |
| Hemoglobin (g/L) | 124.58 ± 24.01 | | 88.45 ± 23.61 | 131.76 ± 16.47 | <0.001 |
| Platelet (×10^9^/L) | 218.00 (174.50, 273.50) | | 310.00 (279.00, 357.00) | 200.00 (169.75, 244.50) | <0.001 |
| Lymphocyte (×10^9^/L) | 1.50 (1.20, 1.85) | | 1.20 (0.85, 1.40) | 1.60 (1.30, 1.90) | <0.001 |
| Short-term complications (n, %) |  | |  |  | 0.779 |
| Intestinal obstruction | 3 (5.77) | | 0 (0.00) | 3 (6.98) |  |
| Cutaneous infection | 14 (26.92) | | 4 (44.44) | 10 (23.26) |  |
| Abdominal bloating | 8 (15.38) | | 1 (11.11) | 7 (16.28) |  |
| Abdominal infection | 8 (15.38) | | 1 (11.11) | 7 (16.28) |  |
| Pulmonary infection | 7 (13.46) | | 1 (11.11) | 6 (13.95) |  |
| Fungal infection | 2 (3.85) | | 1 (11.11) | 1 (2.33) |  |
| Urinary tract infection | 2 (3.85) | | 0 (0.00) | 2 (4.65) |  |
| Fat liquefaction | 2 (3.85) | | 0 (0.00) | 2 (4.65) |  |
| Abdominal haemorrhage | 1 (1.92) | | 1 (11.11) | 0 (0.00) |  |
| Upper respiratory infection | 1 (1.92) | | 0 (0.00) | 1 (2.33) |  |
| Biliary fistula | 1 (1.92) | | 0 (0.00) | 1 (2.33) |  |
| Gastroparesis | 1 (1.92) | | 0 (0.00) | 1 (2.33) |  |
| Leakage of milk curd | 1 (1.92) | | 0 (0.00) | 1 (2.33) |  |
| Drainage tube port infection | 1 (1.92) | | 0 (0.00) | 1 (2.33) |  |

*indicated for *P* < 0.05; NRS 2002: Nutritional Risk Screening 2002; BMI: body mass index; ALB: albumin; CRP: C‑reactive protein; PNI: prognostic nutritional index; PLR: platelet‑to‑lymphocyte ratio; NLR: neutrophil‑to‑lymphocyte ratio; AJCC: American Joint Committee on Cancer; RMB: Renminbi (Chinese yuan).

**Supplement.2 Subgroup analyses according to cancer type of PFS**

|  | **Gastric cancer** | | **Colorectal cancer** | |
| --- | --- | --- | --- | --- |
| **Factors** | **HR (95% CI) *P*** | | **HR (95% CI) *P*** | |
| HALP group (High vs Low) | 0.549 (0.211, 1.430) | 0.220 | 0.417 (0.162, 1.071) | 0.069 |
| Postoperative adjuvant therapy (Yes vs No) | 1.353 (0.498, 3.681) | 0.553 | 7.115 (2.097, 24.145) | 0.002* |
| Tumor stage (AJCC) |  | 0.679 |  | 0.152 |
| II vs I | 0.923 (0.285, 2.988) | 0.894 | 0.245 (0.054, 1.106) | 0.067 |
| III vs I | 1.432 (0.414, 4.945) | 0.571 | 0.217 (0.045, 1.057) | 0.059 |

*indicated for *P* < 0.05
